# Autoantibodies against endothelial protein C receptor and integrin αvβ6 predict the development of ulcerative colitis

**DOI:** 10.1007/s00535-025-02263-7

**Published:** 2025-05-15

**Authors:** Motoi Sawahashi, Yoichi Kakuta, Takeo Naito, Soshi Okazaki, Kinuko Ohneda, Masatsugu Orui, Taku Obara, Soichi Ogishima, Kazuki Kumada, Hisaaki Kudo, Fuji Nagami, Atsushi Hozawa, Hideya Iwaki, Hiroshi Nagai, Yusuke Shimoyama, Rintaro Moroi, Hisashi Shiga, Yoshitaka Kinouchi, Tsuyoshi Shirai, Hiroshi Fujii, Atsushi Masamune

**Affiliations:** 1 Division of Gastroenterology, Tohoku University Graduate School of Medicine, 1-1, Seiryo, Aoba-Ku, Sendai, Miyagi 980-8574 Japan; 2 Department of Rheumatology, Tohoku University Hospital, Sendai, Japan; 3 Tohoku Medical Megabank Organization, Tohoku University, Sendai, Japan; 4 Advanced Research Center for Innovations in Next-Generation Medicine, Tohoku University, Sendai, Japan; 5 Division of Disaster Public Health, International Research Institute of Disaster Science, Tohoku University, Sendai, Japan; 6 Student Healthcare Center, Institute for Excellence in Higher Education, Tohoku University, Sendai, Japan; 7 Division of Epidemiology, Tohoku University Graduate School of Medicine, Sendai, Japan

**Keywords:** Anti-EPCR antibody, Anti-integrin αvβ6 antibody, Ulcerative colitis

## Abstract

**Background:**

A method for predicting ulcerative colitis (UC) onset has not been established. Serum autoantibodies have been suggested as potential predictive biomarkers for UC onset. We aimed to validate the risks associated with serological and environmental factors and construct a model for predicting UC development.

**Methods:**

Using the population-based cohort studies (*n* > 83,000), we identified 42 individuals who were diagnosed with UC later in life and compared them with matched healthy controls. We analyzed serum anti-integrin αvβ6 antibody (anti-αvβ6) and anti-endothelial protein C receptor antibody (anti-EPCR) titers, and lifestyle and dietary habits to explore UC onset predictors. The predictive performance of the models was evaluated based on these predictors.

**Results:**

The sensitivity and specificity of anti-EPCR for predicting UC onset were 51.4% and 97.8%, respectively, comparable to those of anti-αvβ6 (52.5% and 97.6%, respectively). The proportion of individuals with insomnia was significantly higher in the preclinical UC group (adjusted odds ratio = 2.14, 95% confidence interval [CI] 1.11–4.04, *p* = 0.019). The predictive performance of anti-EPCR alone was high with an area under the curve (AUC) of 0.89 (95%CI 0.83–0.96), and that of anti-EPCR combined with anti-αvβ6 was even better with an AUC of 0.92 (95%CI 0.87–0.97); the lifestyle model had lower predictive accuracy (AUC = 0.65, 95%CI 0.55–0.74).

**Conclusions:**

Anti-EPCR and anti-αvβ6 each strongly predict UC onset. The combined anti-EPCR and anti-αvβ6 model had stronger predictive performance than the single models.

**Supplementary Information:**

The online version contains supplementary material available at 10.1007/s00535-025-02263-7.

## Introduction

Ulcerative colitis (UC) is an idiopathic, chronic inflammatory disorder of the colonic mucosa [1]. Similarly to other immune-mediated inflammatory diseases, it has been suggested that the onset of UC begins in the preclinical phase, but its preclinical pathology has not yet been elucidated [2, 3]. To predict UC onset and implement early intervention, a comprehensive understanding of the pathways that drive disease onset in the preclinical phase is required [3].

In recent years, it has been suggested that serum antibodies may reflect abnormalities in the immune system from a few years to 10 years before UC diagnosis [4–7]. Kuwada et al. reported anti-integrin αvβ6 antibodies (anti-αvβ6) as novel biomarkers for the specific diagnosis of UC and its disease activity [8]. In 2023, a US cohort study suggested that serum anti-αvβ6 measurement can diagnose UC up to 10 years before onset, and indicated that anti-αvβ6 are useful biomarkers for predicting the development of UC [7].

We recently identified anti-endothelial protein C receptor antibodies (anti-EPCRs) in Takayasu arteritis and showed that anti-EPCR were also associated with UC; they had pathogenic effects owing to their promotion of Th17 differentiation and endothelial activation [9, 10]. Subsequently, we showed that serum anti-EPCR were specifically positive in UC patients without the complication of Takayasu arteritis in Japanese and Western populations [11]. However, it is unclear whether anti-EPCR can be used as a biomarker to predict UC onset.

Previous studies have identified multiple environmental factors as risks for UC, including past smoking, unhealthy diet, and abnormal sleep duration [12–14]. However, few studies have comprehensively assessed the contribution of environmental and other factors to predict UC onset [13]. The Tohoku Medical Megabank (TMM) Project conducted a prospective population-based cohort study in Miyagi and Iwate prefectures [15]. A strength of this cohort study is that biologic specimens and data collected from participants in healthy states prior to disease onset can be examined to identify potential predictors of disease development.

In this study, we utilized blood samples and associated information from the TMM biobank to examine the risk factors for developing UC by integrating autoantibodies and environmental factors. We aimed to construct prediction models for the onset of UC based on these risk factors.

## Methods

### Study design and participants

This study was a nested case–control study comparing individuals with preclinical UC (cases) with healthy individuals (controls). The study population comprised participants from the TMM Community-Based Cohort Study and the TMM Three-Generation Cohort Study in Miyagi Prefecture, Japan [15–17]. We obtained informed consent from all participants to collect their data and samples. The study was approved by the ethics committee of Tohoku University Graduate School of Medicine (2022-1-411).

### Case and control selection

**Fig. 1:**
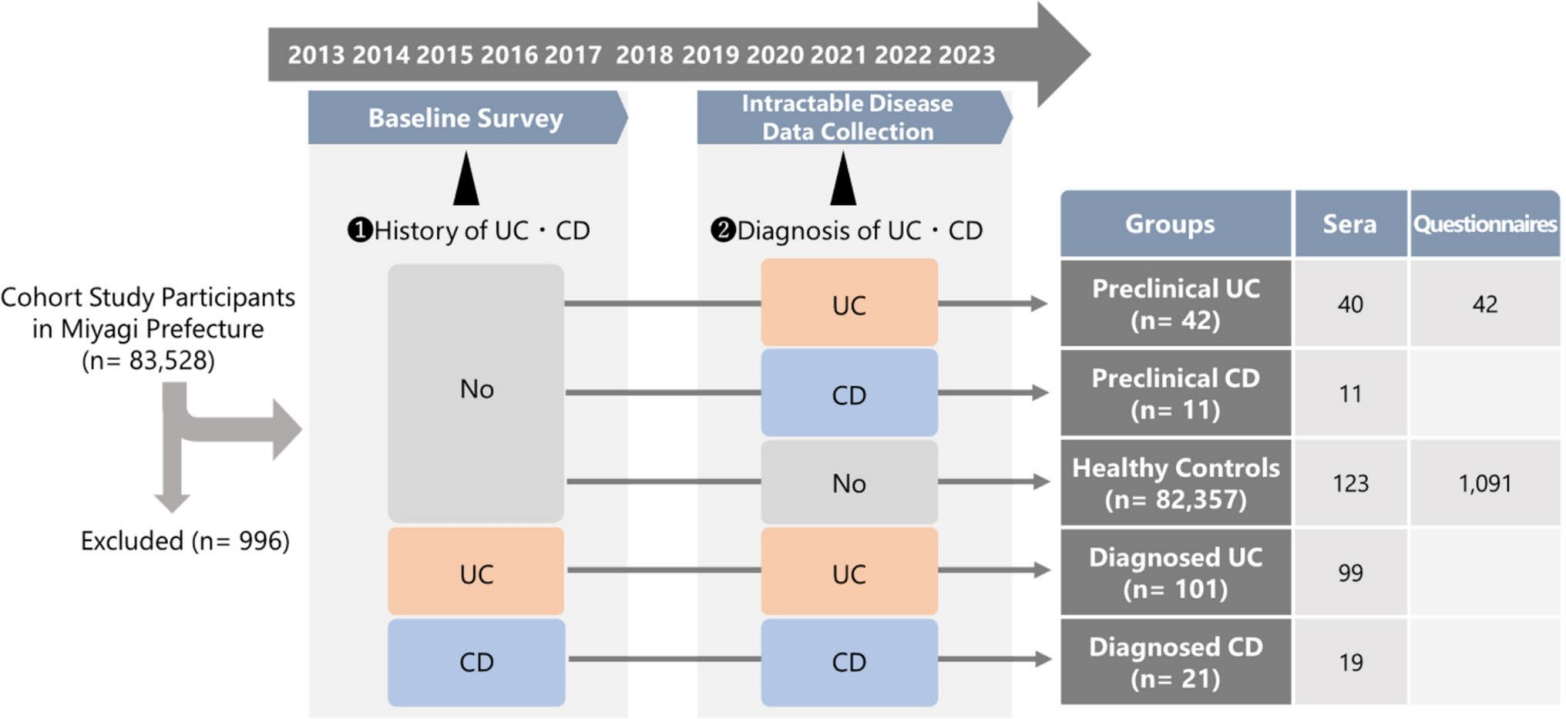
Flowchart of the study. A history of UC or CD was determined based on the information provided about previous diseases during the baseline survey (July 2013 to March 2017). A diagnosis of UC or CD was determined by the information in the national registry of designated intractable diseases from April 2019 to March 2023. Based on the conditions, the participants were extracted and divided into five groups. *UC* ulcerative colitis, *CD* Crohn’s disease, *HCs* healthy controls

Figure 1 is a flowchart of the method used to select study participants. The preclinical UC and Crohn’s disease (CD) groups included individuals who had no history of UC or CD at baseline (July 2013 to March 2017) and were diagnosed with UC and CD, respectively, during the observation period from baseline to July 2023. The diagnosed UC or CD groups included those with a history of UC or CD at baseline. The healthy controls (HCs) were defined as those who had no history of UC or CD at the baseline survey and no diagnosis of UC or CD during the observation period. The healthy group was matched with the preclinical UC group using age, gender, and observation period as covariates. The ratio of cases to controls was 1:1 for serum and 1:10 for questionnaire information for individuals with UC.

A diagnosis of UC or CD was determined by the information in the national registry of designated intractable diseases, which included UC and CD. The information on intractable diseases was collected from the Miyagi prefectural office and the Sendai City office as part of the follow-up survey for the TMM Project. This study utilized the information collected from April 2019 to March 2023. We also collected clinical data from the disease registry available as of 2023, including disease activity (classification of severity [18], International Organization for the Study of Inflammatory Bowel Disease score [19]), disease extent, disease location, refractory disease, and extraintestinal complications.

The exclusion criteria for study participants were as follows: cohort participants in Iwate Prefecture, those with no baseline questionnaire, those with a history of inflammatory bowel disease (IBD) on the baseline survey but no intractable disease information for IBD, and HCs with intractable disease information for a disease other than IBD.

### Measurement of serum autoantibodies

We collected sera (stored at – 80°C) at baseline and during follow-up survey. Anti-αvβ6 and anti-EPCR titers were measured for the preclinical UC and CD groups, diagnosed UC and CD groups, and matched HC group to assess diagnostic performance at baseline.

Serum IgG against integrin αvβ6 was measured using an Anti-Integrin αvβ6 ELISA Kit (Medical & Biological Laboratories, Tokyo, Japan) according to the manufacturer’s protocol. The cutoff value for anti-αvβ6 was set at 2.69 U/mL based on the manufacturer’s recommendation.

For the measurement of serum anti-EPCR, we performed a cell-based assay using flow cytometry. The details of the assay have been reported previously [9]. The cutoff value for anti-EPCR activity was set at 18.0, corresponding to the mean plus three standard deviations (mean + 3SD) of the values in HCs, following the previous study [11]. Samples with non-specific binding patterns were excluded from the analysis because they were not measurable; 50 out of 292 samples (17.1%) were excluded at baseline.

### Evaluation of lifestyle and dietary habits

We used the information on lifestyle and dietary habits at baseline and compared the preclinical UC group with the matched HCs to explore environmental factors related to UC development. For lifestyle factors, we evaluated body mass index, smoking history, drinking history, history of keeping pets (dogs and cats), family history of IBD, exercise habits, and Athens insomnia scale (AIS) [20], Center for Epidemiological Studies Depression [21], and K-6 questionnaire scores [22].

We used a food frequency questionnaire (FFQ) consisting of 138 food and beverage items at baseline to estimate the daily intake of 12 food groups and 17 nutrients. The FFQ is based on the questionnaire used in the Japan Public Health Center-based prospective study, and its validity has been confirmed in previous research [23].

### Statistical analysis

For titers of autoantibodies by disease and clinical subtype, the Wilcoxon rank sum test was used for two-group comparisons, and the Kruskal–Wallis test was used for three-group comparisons. We calculated the sensitivity, specificity, positive likelihood ratio, and negative likelihood ratio to assess the diagnostic performance of autoantibody titers at baseline. We performed regression analysis to examine the correlation between the two autoantibody titers and calculated Spearman’s rank correlation coefficient. The longitudinal comparison of antibody titers was conducted in two ways: (1) between two time points before UC diagnosis (points A and B), and (2) between one time point before and one after UC diagnosis (points C and D). Eleven pairs were compared between points A and B, and ten pairs between points C and D, with only two overlapping cases. The Wilcoxon signed-rank test was used for statistical analysis.

For each lifestyle habit variable, we performed a univariate analysis using Fisher’s exact test and a multivariate analysis using logistic regression analysis, and determined the odds ratio (OR) and 95% confidence interval (CI). For dietary habit factors, logistic regression analysis was performed by dividing daily intake into quartiles (Q1–Q4) for each food category and nutrient, as in Sakamoto et al. [24]. For a single dietary item, a trend test was performed to calculate the p-value for the trend. The residual method was used to adjust the energy intake. We performed receiver operating characteristic (ROC) analysis and calculated the area under the curve (AUC) to assess the diagnostic performance of a model. We used R (4.2.1) software for the analyses. For all tests, *p* ≤ 0.05 was considered statistically significant.

## Results

### Study participants

There were 42 individuals in the preclinical UC group, 101 in the diagnosed UC group, 11 in the preclinical CD group, 21 in the diagnosed CD group, and 82,357 in the HC group, and the observation period was 677,174.4 person-years. In this cohort, the prevalence of UC at the baseline survey was 122/100,000 persons, and the crude incidence of UC was 6.2/100,000 person-years. In the preclinical UC group, the mean age at baseline was 39.1 ± 13.3 years, 47.6% were male, and the median time from baseline to the diagnosis of UC was 4.57 years (Table 1). Anti-αvβ6 and anti-EPCR titers were measured for 40 individuals in the preclinical UC group, 99 in the diagnosed UC group, 11 in the preclinical CD group, 19 in the diagnosed CD group, and 123 matched HCs, excluding 6 participants whose baseline sera were absent. The lifestyle and dietary habit information was analyzed for 42 individuals in the preclinical UC group and 1091 individuals from the matched HCs.

**Table 1:** Baseline characteristics of each group

	Preclinical UC	Diagnosed UC	Preclinical\ CD	Diagnosed\ CD	HCs\ (sera)	HCs\ (questionnaires)
No. individuals	42	101	11	21	123	1,091
Male, *n* (%)	20 (47.6)	35 (34.7)	8 (72.7)	15 (71.4)	57 (46.3)	519 (47.6)
Age at baseline, y, mean (SD)	39.1 (13.3)	46.4 (15.5)	39.5 (9.9)	41.2 (11.4)	39.1 (13.1)	39.0 (13.1)
Time from diagnosis to baseline, y, median [IQR]	− 4.57 [− 6.2– − 2.0]	6.69 [2.9–11.3]	− 1.31 [− 3.6–0]	8.59 [4.1–12.6]	NA	NA
Observation period, y, mean (SD)	7.43 (1.6)	7.72 (1.2)	7.15 (1.1)	8.00 (1.1)	7.63 (1.5)	7.55 (1.5)

*UC* ulcerative colitis, *CD* Crohn’s disease, *HCs* healthy controls, *SD* standard deviation, *IQR* interquartile range, *NA* not applicable

### High positivity rates for anti-αvβ6 and anti-EPCR were confirmed in the preclinical UC group

**Fig. 2:**
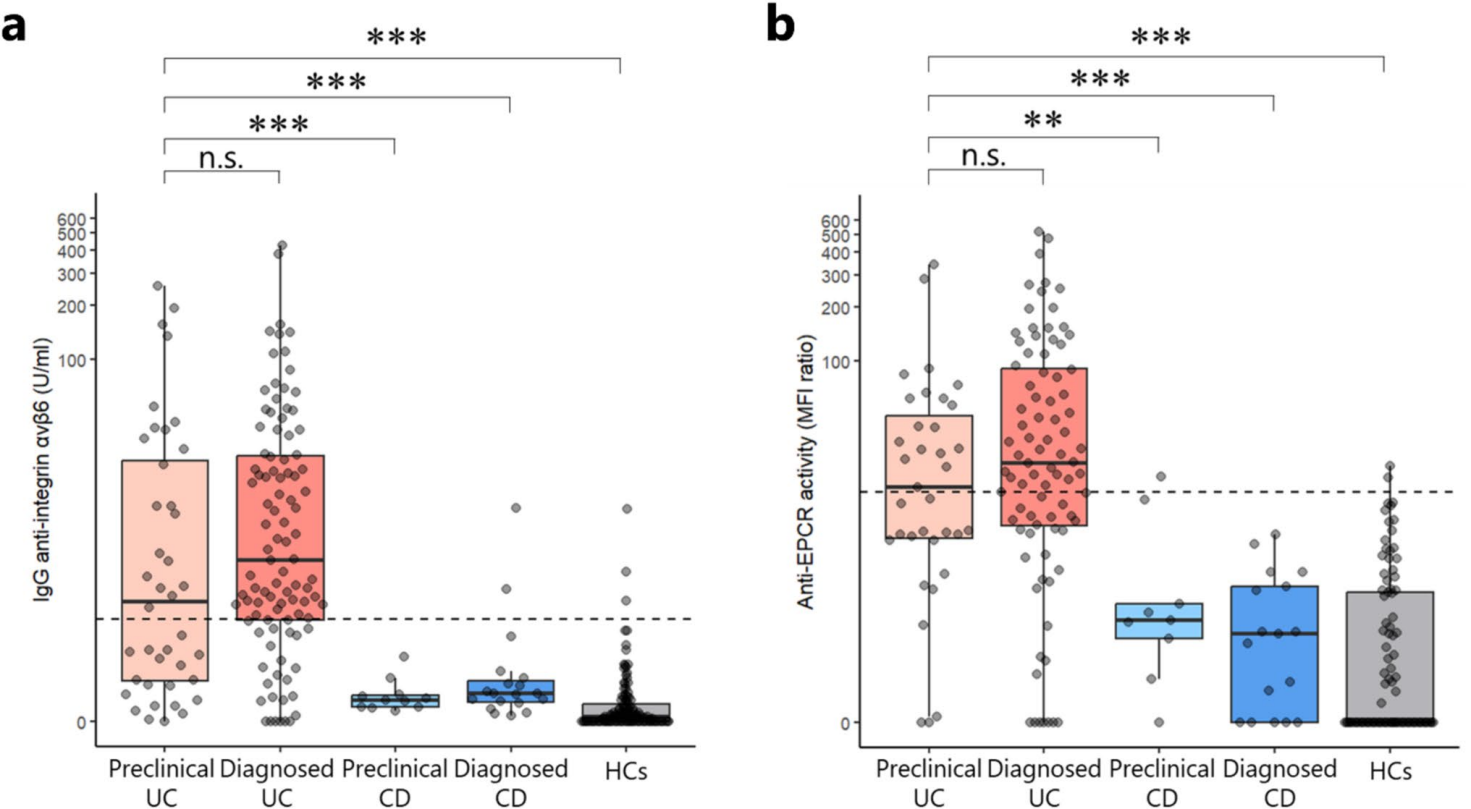
The scatter and box plots illustrate **a** anti-αvβ6 titers and **b** anti-EPCR titers by disease group at baseline. The vertical axis shows each antibody titer on a log(1 + antibody titer) scale. The cutoff value for the antibody titer is indicated by the dotted line. Asterisks indicate levels of statistical significance: *p ≤ 0.05; **p ≤ 0.01; ***p ≤ 0.001 (Wilcoxon signed-rank test). *anti-αvβ6* anti-integrin αvβ6 antibody, *anti-EPCR* anti-endothelial protein C receptor antibody, *MFI* mean fluorescence intensity, *n.s.* not significant, *UC* ulcerative colitis, *CD* Crohn’s disease, *HCs* healthy controls

The positivity rates for anti-αvβ6 and anti-EPCR antibodies were high in both the preclinical UC group and the diagnosed UC group, with anti-αvβ6 positivity rates of 52.5% and 73.7%, respectively, and anti-EPCR positivity rates of 51.4% and 63.6%, respectively (Fig. 2, Supplemental Table 1). Both antibody titers were significantly higher in the preclinical UC group than the preclinical CD group, diagnosed CD group, and HCs. Their sensitivity and specificity for predicting UC were 52.5% and 97.6%, respectively, for anti-αvβ6 and 51.4% and 97.8%, respectively, for anti-EPCR (Table 2).

**Table 2:** Diagnostic performance of anti-αvβ6 and anti-EPCR titers for ulcerative colitis

	Sensitivity	Specificity	LR +	LR −
anti-αvβ6 (Cutoff 2.69 U/ml)				
Preclinical UC	52.5%	97.6%	21.5	0.49
Diagnosed UC	73.7%	97.6%	30.2	0.27
anti-EPCR (Cutoff 18)				
Preclinical UC	51.4%	97.8%	23.9	0.50
Diagnosed UC	63.6%	97.8%	29.6	0.37

*UC* ulcerative colitis, *anti-αvβ6* anti-integrin αvβ6 antibody, *anti-EPCR* anti-endothelial protein C receptor antibody,* LR +* positive likelihood ratio, *LR–* negative likelihood ratio

Based on the combination of positive and negative results for both antibodies, patients were divided into four groups: double-positive, anti-αvβ6 single-positive, anti-EPCR single-positive, and double-negative (Table 3). Double-positivity was more specific for individuals with UC (preclinical UC group: 31.4%; diagnosed UC group: 51.1%; preclinical and diagnosed CD groups, and HCs: 0%) than single-positivity.

**Table 3:** Combination of positive and negative results for anti-αvβ6 and anti-EPCR

Anti-αvβ6/anti-EPCR		Positive/Positive		Positive/Negative		Negative/Positive		Negative/Negative	
*N*		*n*	%	*n*	%	*n*	%	*n*	%
Preclinical UC	35	11	31.4%	7	20%	7	20%	10	28.6%
Diagnosed UC	88	45	51.1%	21	23.9%	11	12.5%	11	12.5%
Preclinical CD	9	0	0%	0	0%	1	11.1%	8	88.9%
Diagnosed CD	17	0	0%	2	11.8%	0	0%	15	88.2%
Healthy controls	93	0	0%	3	3.2%	2	2.2%	88	94.6%

*UC* ulcerative colitis, *CD* Crohn’s disease, *anti-αvβ6* anti-integrin αvβ6 antibody, *anti-EPCR* anti-endothelial protein C receptor antibody

### Anti-EPCR predicted the onset of UC as accurately as anti-αvβ6, and the combination model demonstrated even greater predictive performance

**Fig. 3:**
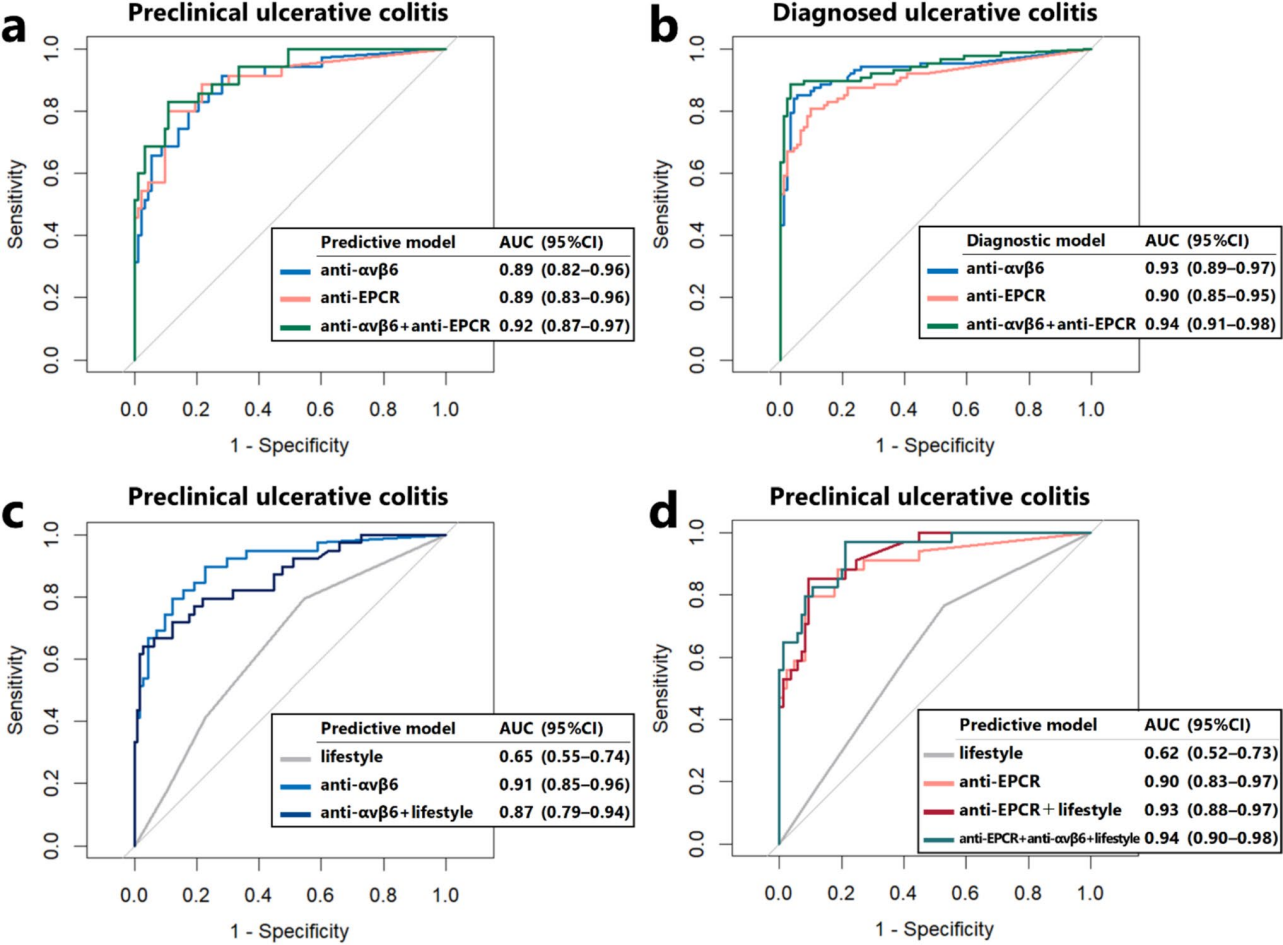
Receiver operating characteristic (ROC) analysis of models based on anti-αvβ6, anti-EPCR, lifestyle factors, and their combinations. The ROC curves represent the performance of (**a**) autoantibody models for the preclinical UC group, (**b**) autoantibody models for the diagnosed UC group, (**c**) models that include anti-αvβ6 and lifestyle for 153 individuals (39 in the preclinical UC group and 114 of the HCs), and (**d**) models that include anti-EPCR and lifestyle for 119 individuals (34 in the preclinical UC group and 85 of the HCs). *anti-αvβ6* anti-integrin αvβ6 antibody, *anti-EPCR* anti-endothelial protein C receptor antibody, *AUC* area under the curve, *CI* confidence interval

Anti-αvβ6 and anti-EPCR titers in the single and combination models were evaluated for the preclinical UC and diagnosed UC groups (Fig. 3a, b). The predictive performance of anti-αvβ6 for UC onset had an AUC of 0.89 (95%CI 0.82–0.96); that of anti-EPCR had an AUC of 0.89 (95%CI 0.83–0.96). The combination model with anti-αvβ6 and anti-EPCR (AUC = 0.92, 95%CI 0.87–0.97) showed higher predictive performance than the single models, although the difference did not reach statistical significance (versus anti-αvβ6, *p* = 0.38; versus anti-EPCR, *p* = 0.070). The UC diagnostic performance of anti-αvβ6 had an AUC of 0.93 (95%CI 0.89–0.97) and that of anti-EPCR had an AUC of 0.90 (95%CI 0.85–0.95), whereas the AUC for the anti-αvβ6 and anti-EPCR combination model increased to 0.94 (95%CI 0.91–0.98).

### Positivity rates for anti-αvβ6 and anti-EPCR are high in active UC cases, and titers of both autoantibodies are positively correlated

We analyzed autoantibody titers according to clinical subtype (Supplemental Tables 2, 3; Supplemental Figs. 1, 2). In the preclinical UC group, anti-αvβ6 titers were significantly higher in cases with moderate activity than in those with mild activity (*p* = 0.050), and positivity rates for both antibodies were also higher in cases with moderate activity (anti-αvβ6: mild 12.5%, moderate 57.1%; anti-EPCR: mild 37.5%, moderate 58.3%). There were no other clinical subtype factors relevant to autoantibody titers.

We next examined the correlation between anti-αvβ6 and anti-EPCR (Supplemental Fig. 3). We found a significant correlation between anti-αvβ6 and anti-EPCR titers in preclinical and diagnosed UC groups (preclinical UC group: *r* = 0.47, *p* = 0.004; diagnosed UC group: *r* = 0.42, *p* < 0.001), but not in the non-UC group.

### The positivity rates for anti-αvβ6 and anti-EPCR increased as diagnosis approached in the preclinical UC phase

We examined longitudinal antibody titers in the preclinical UC group (Supplemental Table 4, Supplemental Fig. 4). First, we compared 11 pairs of samples between two time points (points A and B) before UC diagnosis. The positivity rates for both antibodies were higher at the point closer to diagnosis (anti-αvβ6: 36.4% to 63.6%; anti-EPCR: 50.0% to 80.0%), and anti-EPCR titers were significantly higher at the point closer to diagnosis (*p* = 0.014). No significant changes in antibody titers were observed between the two time points before and after diagnosis.

### Insomnia was associated with UC onset among the environmental factors investigated

The analysis of lifestyle habits was performed using data from 41 individuals in the preclinical UC group, as lifestyle data were lacking for 1 individual (Table 4). The percentage of patients with an AIS ≥ 6 points, corresponding to insomnia, was significantly higher in the preclinical UC group than among the HCs (41.5% and 24.7%, respectively; crude OR = 2.16, 95%CI 1.07–4.27, *p* = 0.025). The percentage of patients with a history of smoking was higher in the preclinical UC group, but the difference was not statistically significant (58.5% and 44.6%, respectively; crude OR = 1.68, 95%CI 0.85–3.37, *p* = 0.11). Multivariate logistic regression was performed for AIS ≥ 6 points, smoking history, and drinking history, revealing that AIS ≥ 6 points (adjusted OR = 2.14, 95%CI 1.11–4.04, *p* = 0.019) was significantly associated with preclinical UC.

**Table 4:** Univariate and multivariate analyses for lifestyle factors

		Preclinical UC	Healty Controls	Univariate		Multivariate	
		*n* (%)	*n* (%)	Odds ratio (95%CI)	*p*-value	Odds ratio (95%CI)	*p*-value
*N*		41 (100)	1,091 (100)				
Body mass index	18.5 ≥ , < 25	31 (75.6)	731 (70.4)	(reference)			
	≥ 25	7 (17.1)	220 (21.2)	0.75 (0.28–1.77)	0.69		
	< 18.5	3 (7.3)	87 (8.4)	0.81 (0.16–2.69)	1		
	NA	0 (0)	53 (4.9)				
Smoking history	No	17 (41.5)	579 (53.1)	(reference)			
	Yes	24 (58.5)	487 (44.6)	1.68 (0.85–3.37)	0.11	1.85 (0.96–3.63)	0.067
	Former smoker	15 (36.6)	282 (25.8)	1.81 (0.83–3.92)	0.12		
	Current smoker	9 (22.0)	205 (18.8)	1.49 (0.58–3.61)	0.37		
	NA	0 (0)	53 (4.9)				
Drinking history	No	19 (46.3)	423 (38.8)	(reference)			
	Yes	22 (53.7)	628 (57.6)	0.78 (0.40–1.54)	0.52	0.69 (0.36–1.34)	0.27
	Former drinker	2 (4.9)	96 (8.8)	0.46 (0.05–1.98)	0.40		
	Current drinker	20 (48.8)	532 (48.8)	0.84 (0.42–1.68)	0.62		
	NA	0 (0)	40 (3.7)				
Keeping pets	No	31 (75.6)	857 (78.6)	(reference)			
	Yes	10 (24.4)	234 (21.4)	1.18 (0.51–2.52)	0.70		
IBD family history	No	41 (100.0)	1,088 (99.7)	(reference)			
	Yes	0 (0.0)	3 (0.3)	0(0–65.3)	1		
Exercise habits	< 10MET-h/wk	32 (78.0)	767 (70.3)	(reference)			
	≥ 10MET-h/wk	9 (22.0)	324 (29.7)	0.67 (0.28–1.45)	0.38		
AIS	< 6	24 (58.5)	790 (75.3)	(reference)			
	≥ 6	17 (41.5)	259 (24.7)	**2.16 (1.07–4.27)**	**0.025**	**2.14 (1.11–4.04)**	**0.019**
	NA	0 (0)	42 (3.8)				
CES-D	< 16	31 (75.6)	784 (78.2)	(reference)			
	≥ 16	10 (24.4)	219 (21.8)	1.15 (0.50–2.46)	0.70		
	NA	0 (0)	88 (8.1)				
K-6	< 10	34 (82.9)	898 (85.3)	(reference)			
	≥ 10	7 (17.1)	155 (14.7)	1.19 (0.44–2.80)	0.65		
	NA	0 (0)	38 (3.5)				

Bold values indicate statistically significant associations (*p* < 0.05). UC, ulcerative colitis; IBD, inflammatory bowel disease; AIS, Athens insomnia scale; CES-D, Center for Epidemiological Studies Depression; MET, metabolic equivalent of task; NA, not available; CI, confidence interval

The dietary habits analysis was conducted with data from 36 individuals in the preclinical UC group, as dietary data were lacking for 6 individuals in the group. No nutrients or food categories were found to be significantly associated with preclinical UC (Supplemental Tables 5, 6).

### Prediction model for UC development based on serum autoantibodies and environmental factors

We developed a regression model based on the lifestyle factors of insomnia and smoking history to evaluate its predictive performance for UC development. We performed ROC analysis for the lifestyle model, serum autoantibody model (anti-αvβ6 and anti-EPCR), and the combined model (Fig. 3c, d). The lifestyle single model (AUC = 0.65, 95%CI 0.55–0.74) had a significantly lower predictive test performance than the anti-αvβ6 and anti-EPCR single models (*p* < 0.001 and *p* < 0.001, respectively). Combination with the lifestyle model slightly improved the predictive performance of the anti-EPCR single model (AUC 0.90–0.93), but not the anti-αvβ6 single model (AUC 0.91 to 0.87).

## Discussion

We investigated anti-αvβ6, anti-EPCR, and lifestyle and dietary habits as predictive factors for UC development. Here, we demonstrated for the first time that anti-EPCR predicts UC development approximately 4.5 years before diagnosis with the same high accuracy as anti-αvβ6. Our results also indicated that a model combining titers of the two autoantibodies has higher predictive ability than either of the single models. The model based on lifestyle and dietary habits had lower predictive accuracy than the autoantibody models.

A strength of this study is the use of samples and data from the largest prospective, population-based cohort in Japan. To analyze causal relationships with disease onset, we integrated cohort study data with diagnostic information from the national registry of designated intractable diseases. To the best of our knowledge, this is the first study to simultaneously analyze both serum antibody levels and environmental factors in relation to UC onset.

Anti-αvβ6 and anti-EPCR are the most accurate biomarkers of UC development at present, compared with the circulating antibodies used in past studies. In 2005, Israeli et al. first reported that perinuclear antineutrophil cytoplasmic antibody (pANCA) levels can predict the onset of UC several years in advance of diagnosis [4]. Further studies found that combined models of serological markers including pANCA enhanced predictive ability; however, they were not sufficiently accurate [5, 6]. Importantly, Livanos et al. showed that anti-αvβ6 titers can predict the onset of UC with the highest accuracy, with AUCs ranging from 0.79 at 10 years before diagnosis to 0.89 at 2 years before diagnosis [7]. Our results indicate that anti-EPCR, as well as anti-αvβ6, is likely to be among the most promising predictive markers for UC development. Both anti-αvβ6 and anti-EPCR exhibit high specificity for UC, however, there are some differences between the two antibodies. For instance, anti-EPCR are also detected in patients with Takayasu’s arteritis [9], whereas anti-αvβ6 are detected in patients with primary sclerosing cholangitis [25]. Combining these two antibodies may increase sensitivity and enhance the utility of the test.

Our results suggested that both anti-αvβ6 and anti-EPCR may be markers that reflect UC activity from the preclinical phase to post-diagnosis. The positivity rates for both antibodies before UC diagnosis were high in active cases after UC diagnosis. This finding aligns with those of previous studies that reported anti-αvβ6 and anti-EPCR titers correlate with disease activity scores in diagnosed UC cases [8, 11]. We were unable to compare the clinical data over the same period as the autoantibody titers; however, our study provides novel evidence that autoantibody levels in the preclinical phase of UC could potentially predict disease activity after disease onset. In a longitudinal study of two points before UC diagnosis, the positivity rates for these antibodies increased as the time of UC diagnosis approached. As the first attack of UC approaches, these autoantibody titers may increase, reflecting the progression of disease activity.

The mechanism by which anti-EPCR contribute to the development of UC has not yet been fully elucidated; however, some studies have suggested that EPCR is involved in the pathogenesis of IBD [10, 26, 27]. EPCR is expressed on the endothelium and other cell types, including intestinal epithelial cells [27]. EPCR plays a role in maintaining intestinal barrier function and exerting anti-inflammatory effects via the protein C pathway [27, 28]. EPCR expression is reduced in patients with IBD [26], and EPCR-deficient mice are highly susceptible to experimental colitis [27]. These findings suggest that the inhibitory effect of anti-EPCR on EPCR may contribute to UC pathogenesis. The pre-diagnostic autoimmune changes in anti-EPCR observed in this study may reflect dysregulated immune responses preceding clinical disease [2, 3].

Our analysis of lifestyle factors suggested that insomnia increases the risk of UC development. In a Japanese prospective study, UC relapse rate was high in individuals who reported poor sleep, suggesting that chronic poor sleep is associated with UC disease activity [29]. A US prospective cohort study reported that unusual sleep duration was associated with UC development [30]. These findings support the role of insomnia as an important precursor to UC onset. Some experiments have shown that poor sleep can affect the immune system [31, 32]. Further investigation is needed to understand the underlying mechanisms by which insomnia contributes to UC development.

We did not detect any obvious dietary factors that affected the risk of developing UC. One possible reason is that this study had a small sample size for individuals in the preclinical UC group, compared with previous studies [13, 14, 24]. Lifestyle and dietary habits may have a relatively small impact on UC development years later, considering that the predictive model based on lifestyle and dietary habits is far less accurate than those based on autoantibodies.

This study has several limitations. First, the samples for the preclinical UC group and matched healthy controls were limited, which may have affected the precise estimation of diagnostic performance. Second, the UC prediction diagnostic model, which incorporates autoantibodies and lifestyle factors, has not been externally validated. Future large-scale studies are needed to measure the autoantibody titers in a broader population, evaluate predictive diagnostic performance more accurately, and determine appropriate cutoff values. Third, there are limitations to the scope of available information. In this study, the diagnoses of UC and CD during the observation period were determined based on intractable disease registration data provided by the local government. Therefore, if individuals moved out of the region, their diagnoses may not have been captured. Finally, in the measurement of EPCR antibodies, a proportion of samples in all groups could not be analyzed due to non-specific binding pattern. To date, no specific patient background factors have been identified among individuals showing this pattern. The excluding of these samples may introduce selection bias. Future efforts should focus on developing a more stable and reliable measurement system for anti-EPCR.

In conclusion, we found that anti-EPCR titers can predict UC onset with the same high accuracy as anti-αvβ6 titers, and a model combining titers of these autoantibodies can predict disease onset with even greater accuracy, which could facilitate prevention and early intervention in future.

## Supplementary Information

Below is the link to the electronic supplementary material.

Supplementary file1 (DOCX 1025 KB)

Supplementary file2 (DOCX 64 KB)

## Data Availability

The derived data are available from the authors upon reasonable request and with the permission of the TMM Biobank.

## Abbreviations

AIS: Athens insomnia scale
Anti-αvβ6: Anti-integrin αvβ6 antibody
Anti-EPCR: Anti-endothelial protein C receptor antibody
AUC: Area under the curve
CD: Crohn’s disease
CI: Confidence interval
FFQ: Food frequency questionnaire
IBD: Inflammatory bowel disease
IQR: Interquartile range
OR: Odds ratio
pANCA: Perinuclear antineutrophil cytoplasmic antibody
ROC: Receiver operating characteristic
SD: Standard deviation
Th: T helper cell
TMM: Tohoku Medical Megabank
UC: Ulcerative colitis
