# Supplementary material for: Autoantibodies against endothelial protein C receptor and integrin αvβ6 predict the development of ulcerative colitis
Source: J Gastroenterol. 2025 May 15;60(9):1108–17. doi: 10.1007/s00535-025-02263-7 (PMC12378132; doi:10.1007/s00535-025-02263-7)
Supplement: Supplementary file 1 — Supplementary file1 (DOCX 1025 KB) [file 535_2025_2263_MOESM1_ESM.docx]

**Supplemental Fig. 1**

**
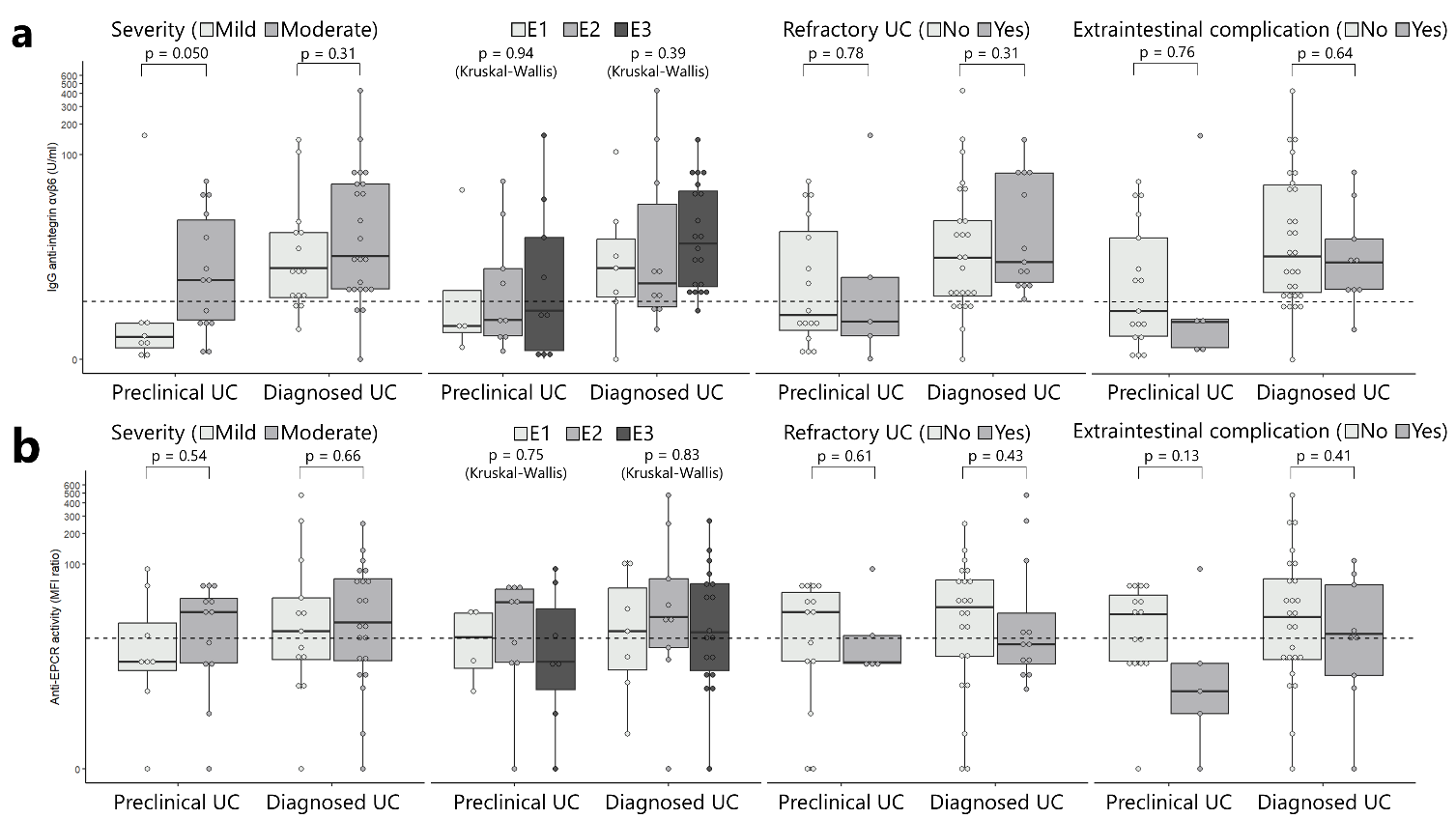
**The scatter and box plots illustrate the (a) anti-αvβ6 titers and (b) anti-EPCR titers of the preclinical and diagnosed ulcerative colitis (UC) groups, stratified by clinical subtypes as of 2023. Clinical subtypes are categorized according to the classification of severity, disease extent (E1, proctitis; E2, left-sided colitis; and E3, pancolitis), refractory UC, and extraintestinal complication. p-values were calculated using the Wilcoxon signed-rank test or the Kruskal–Wallis test. anti-αvβ6, anti-integrin αvβ6 antibody; anti-EPCR, anti-endothelial protein C receptor antibody; MFI, mean fluorescence intensity

**Supplemental Fig. 2**


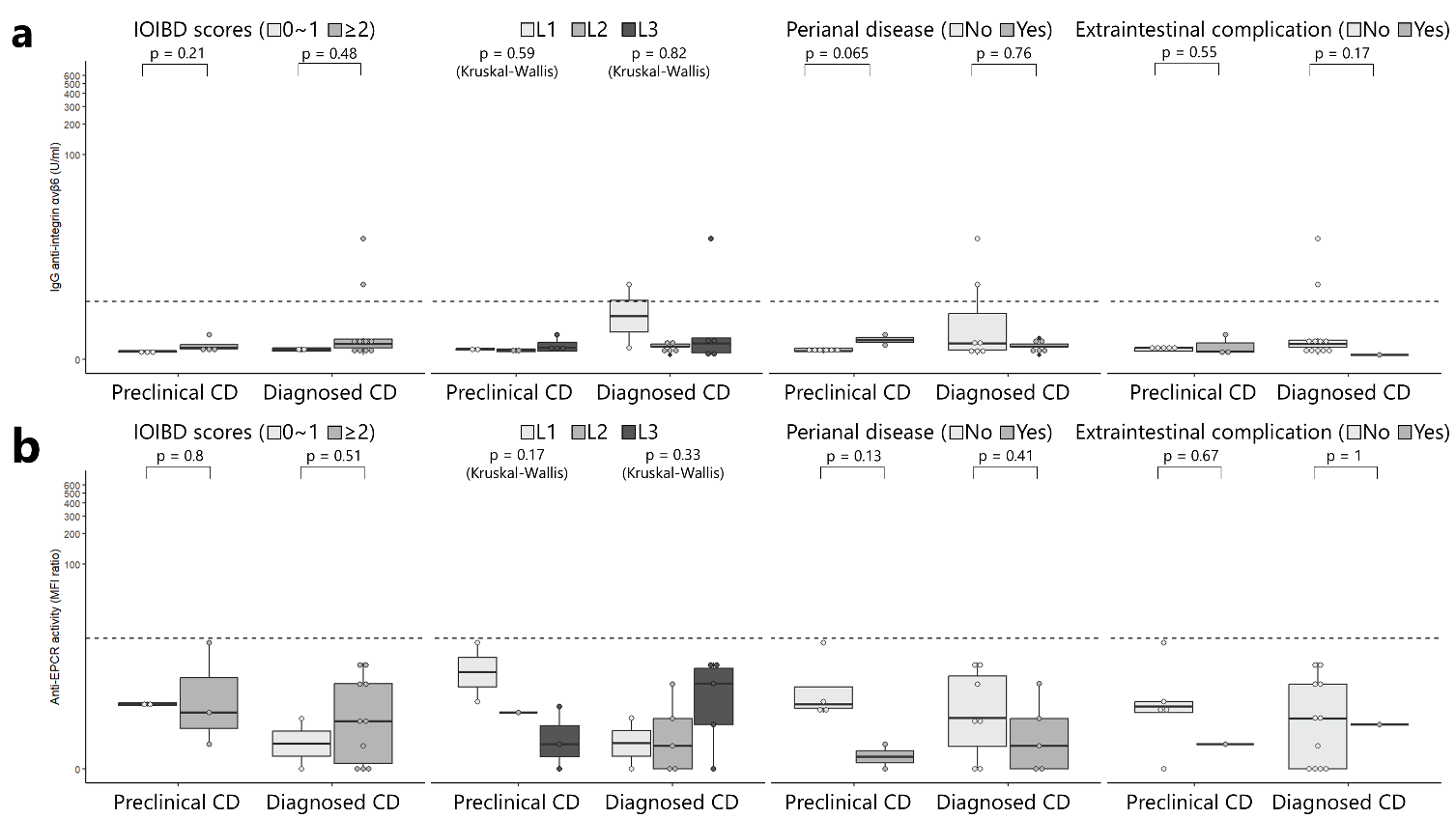
The scatter and box plots illustrate the (a) anti-αvβ6 titers and (b) anti-EPCR titers of the preclinical and diagnosed Crohn’s disease (CD) groups, stratified by clinical subtypes as of 2023. Clinical subtypes are categorized according to International Organization for the Study of Inflammatory Bowel Disease (IOIBD) scores, disease location (L1, ileal; L2, colonic; and L3, ileocolonic), perianal disease, and extraintestinal complication. p-values were calculated using the Wilcoxon signed-rank test or the Kruskal–Wallis test. anti-αvβ6, anti-integrin αvβ6 antibody; anti-EPCR, anti-endothelial protein C receptor antibody; MFI, mean fluorescence intensity

**Supplemental Fig. 3**


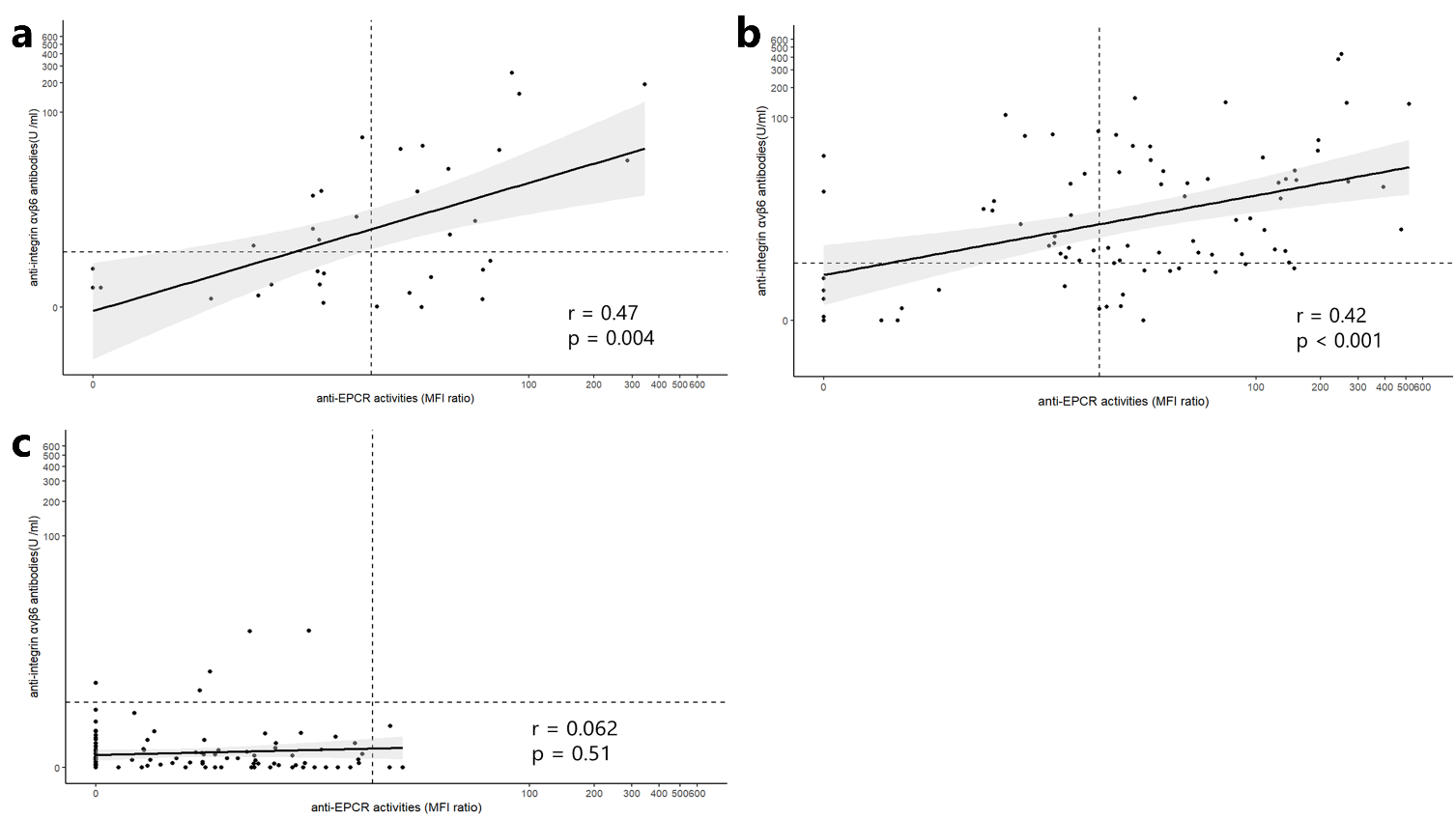
The scatterplots illustrate the correlation between the titers of anti-αvβ6 and anti-EPCR in the (a) preclinical UC group, (b) diagnosed UC group, (c) non-UC group (including those with preclinical CD and diagnosed CD, and HCs). The cutoff value for the antibody titer is indicated by the dotted line. The unbroken straight lines are regression lines; r is the correlation coefficient. p-values are from the regression analysis. anti-αvβ6, anti-integrin αvβ6 antibody; anti-EPCR, anti-endothelial protein C receptor antibody; MFI, mean fluorescence intensity; UC, ulcerative colitis; CD, Crohn’s disease; HCs, healthy controls

**Supplemental Fig. 4**


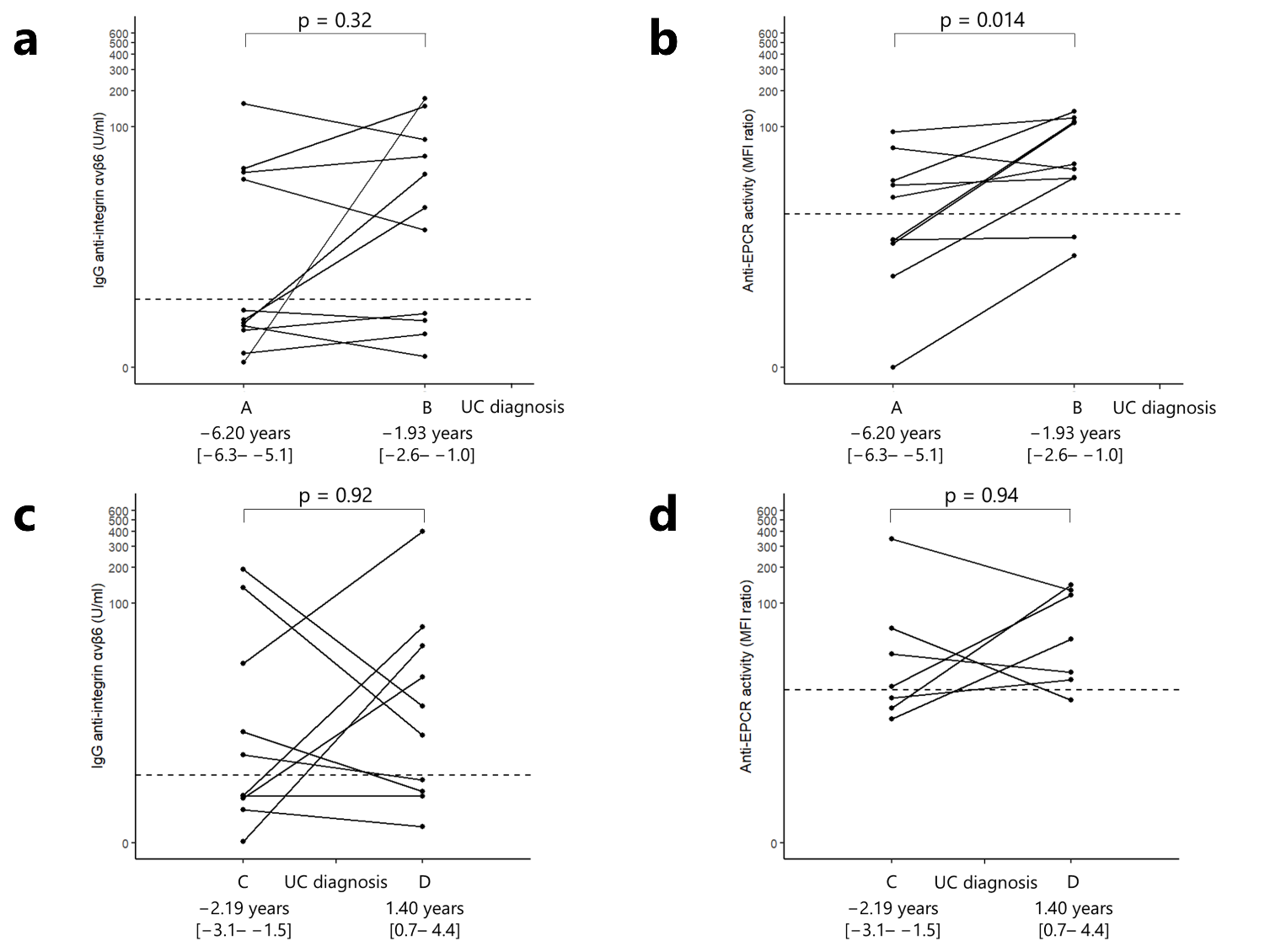
Longitudinal autoantibody titers between two sets of time points. Graphs for (a) anti-αvβ6 and (b) anti-EPCR show the comparison of antibody titers at time points A and B before diagnosis. Graphs for (c) anti-αvβ6 and (d) anti-EPCR show the comparison of antibody titers at time points C (before diagnosis) and D (after diagnosis). The cutoff value for the antibody titer is indicated by the dotted line. p-values are from the Wilcoxon signed-rank sum test. anti-αvβ6, anti-integrin αvβ6 antibody; anti-EPCR, anti-endothelial protein C receptor antibody; MFI, mean fluorescence intensity
