# Supplementary material for: Autoantibodies against endothelial protein C receptor and integrin αvβ6 predict the development of ulcerative colitis
Source: J Gastroenterol. 2025 May 15;60(9):1108–17. doi: 10.1007/s00535-025-02263-7 (PMC12378132; doi:10.1007/s00535-025-02263-7)
Supplement: Supplementary file 2 — Supplementary file2 (DOCX 64 KB) [file 535_2025_2263_MOESM2_ESM.docx]

**Supplementary Table 1. Serum autoantibody titers and positivity at baseline**

|  | anti-αvβ6 | | |  | anti-EPCR | | |  |
| --- | --- | --- | --- | --- | --- | --- | --- | --- |
|  | n | Titer (U/ml, median [IQR]) | Positivity (%) |  | n | Titer (MFI ratio, median [IQR]) | Positivity (%) | |
| **Preclinical UC** | 40 | 3.6 [0.68–26.8] | 52.5 |  | 35 | 19.2 [9.5–49.5] | 51.4 | |
| **Diagnosed UC** | 99 | 6.8 [2.7–28.4] | 73.7 |  | 88 | 29.5 [12.0–113] | 63.6 | |
| **Preclinical CD** | 11 | 0.31 [0.21–0.40] | 0 |  | 9 | 2.7 [1.9–3.6] | 11.1 | |
| **Diagnosed CD** | 19 | 0.43 [0.28–0.68] | 10.5 |  | 17 | 2.1 [0–4.6] | 0 | |
| **Healthy controls** | 123 | 0.06 [0–0.25] | 2.4 |  | 93 | 0 [0–4.3] | 2.2 | |

UC, ulcerative colitis; CD, Crohn’s disease; anti-αvβ6, anti-integrin αvβ6 antibody; anti-EPCR, anti-endothelial protein C receptor antibody; MFI, mean fluorescence intensity; IQR, interquartile range

**Supplementary Table 2. Clinical information on intractable diseases in 2023**

1. **Clinical subtypes of UC groups**

|  |  | **Preclinical UC** | **Diagnosed UC** |
| --- | --- | --- | --- |
| No. of individuals with data available | | 22 | 38 |
| Classification of severity |  |  |  |
| Mild | n (%) | 8 (36.4) | 15 (39.5) |
| Moderate | n (%) | 14 (63.6) | 23 (60.5) |
| Severe | n (%) | 0 (0) | 0 (0) |
| Disease Extent |  |  |  |
| Proctitis | n (%) | 4 (18.2) | 7 (18.4) |
| Left-sided colitis | n (%) | 9 (40.9) | 10 (26.3) |
| Pancolitis | n (%) | 9 (40.9) | 21 (55.2) |
| Refractory UC |  |  |  |
| No | n (%) | 16 (72.7) | 25 (65.8) |
| Steroid-dependent | n (%) | 5 (22.7) | 10 (26.3) |
| Steroid-refractory | n (%) | 0 (0) | 2 (5.3) |
| NA | n (%) | 1 (4.5) | 1 (2.6) |
| Extraintestinal complication | |  |  |
| No | n (%) | 17 (77.3) | 29 (76.3) |
| Joints | n (%) | 1 (4.5) | 4 (10.5) |
| Skin | n (%) | 2 (9.1) | 3 (7.9) |
| Eyes | n (%) | 0 (0) | 1 (2.6) |
| Primary sclerosing cholangitis | n (%) | 0 (0) | 0 (0) |
| Autoimmune pancreatitis | n (%) | 0 (0) | 0 (0) |
| IgG4-related disease | n (%) | 0 (0) | 0 (0) |
| Growth impairment | n (%) | 0 (0) | 0 (0) |
| Osteoporosis | n (%) | 0 (0) | 0 (0) |
| Others | n (%) | 1 (4.5) | 1 (2.6) |

1. **Clinical subtypes of CD groups**

|  |  | **Preclinical CD** | **Diagnosed CD** |
| --- | --- | --- | --- |
| No. of individuals with data available | | 8 | 12 |
| IOIBD score | median [IQR] | 2.0 [0.5–3.0] | 2.0 [2.0–2.0] |
| 0–1 | n (%) | 3 (37.5) | 2 (16.7) |
| ≥2 | n (%) | 4 (50.0) | 10 (83.3) |
| NA | n (%) | 1 (12.5) | 0 (0) |
| Disease Location |  |  |  |
| Ileal | n (%) | 2 (25.0) | 2 (16.7) |
| Colonic | n (%) | 2 (25.0) | 5 (41.7) |
| Ileocolonic | n (%) | 4 (50.0) | 5 (41.7) |
| Upper gastrointestinal disease | |  |  |
| Yes | n (%) | 1 (12.5) | 2 (16.7) |
| No | n (%) | 7 (87.5) | 10 (83.3) |
| Perianal disease |  |  |  |
| Yes | n (%) | 2 (25.0) | 5 (41.7) |
| No | n (%) | 6 (75.0) | 7 (58.3) |
| Extraintestinal complication | |  |  |
| No | n (%) | 5 (62.5) | 11 (91.7) |
| Joints | n (%) | 0 (0) | 1 (8.3) |
| Skin | n (%) | 0 (0) | 0 (0) |
| Eyes | n (%) | 0 (0) | 0 (0) |
| Primary sclerosing cholangitis | n (%) | 0 (0) | 0 (0) |
| Autoimmune pancreatitis | n (%) | 1 (12.5) | 0 (0) |
| IgG4-related disease | n (%) | 0 (0) | 0 (0) |
| Growth impairment | n (%) | 0 (0) | 0 (0) |
| Osteoporosis | n (%) | 0 (0) | 0 (0) |
| Others | n (%) | 2 (25.0) | 0 (0) |

UC, ulcerative colitis; CD, Crohn’s disease; NA, not available; IOIBD, International Organization for the Study of Inflammatory Bowel Disease; IgG4, immunoglobulin G4; IQR, interquartile range

**Supplementary Table 3. Antibody titers and positivity stratified by clinical subtype of UC**

1. **Preclinical UC group**

|  | **Preclinical UC group** (n= 22) | | | | | | |
| --- | --- | --- | --- | --- | --- | --- | --- |
|  | anti-αvβ6 | | |  | anti-EPCR | | |
|  | Titer(U/ml, median[IQR]) | p-value | Positivity(%) |  | Titer(MFI ratio, median[IQR]) | p-value | Positivity(%) |
| Classification of severity | | **0.050** |  |  |  | 0.54 |  |
| Mild | 0.65 [0.29–1.25] |  | 1/8 (12.5) |  | 10.3 [8.5–29.6] |  | 3/8 (37.5) |
| Moderate | 4.97 [1.45–22.7] |  | 8/14 (57.1) |  | 33.3 [10.0–46.1] |  | 7/12 (58.3) |
| Disease extent |  | 0.94 |  |  |  | 0.75 |  |
| Proctitis | 1.14 [0.87–12.0] |  | 1/4 (25.0) |  | 21.1 [9.1–32.6] |  | 2/4 (50.0) |
| Left-sided colitis | 1.43 [0.71–6.74] |  | 4/9 (44.4) |  | 42.0 [10.0–56.3] |  | 5/9 (55.6) |
| Pancolitis | 1.98 [0.22–14.6] |  | 4/9 (44.4) |  | 10.2 [5.9–42.6] |  | 3/7 (42.9) |
| Refractory UC |  | 0.78 |  |  |  | 0.61 |  |
| No | 1.73 [0.94–17.3] |  | 7/16 (43.8) |  | 33.3 [10.3–52.9] |  | 8/14 (57.1) |
| Yes | 1.35 [0.71–5.37] |  | 2/5 (40.0) |  | 10.0 [9.8–19.2] |  | 2/5 (40.0) |
| Extraintestinal complication | | 0.76 |  |  |  | 0.13 |  |
| No | 1.98 [0.71–14.6] |  | 8/17 (47.1) |  | 31.8 [10.4–49.5] |  | 9/15 (60.0) |
| Yes | 1.35 [0.32–1.48] |  | 1/5 (20.0) |  | 4.74 [2.5–9.8] |  | 1/5 (20.0) |

1. **Diagnosed UC group**

|  | **Diagnosed UC group** (n= 38) | | | | | | |
| --- | --- | --- | --- | --- | --- | --- | --- |
|  | anti-αvβ6 | | |  | anti-EPCR | | |
|  | Titer(U/ml, median[IQR]) | p-value | Positivity(%) |  | Titer(MFI ratio, median[IQR]) | p-value | Positivity(%) |
| Classification of severity | | 0.31 |  |  |  | 0.66 |  |
| Mild | 6.82 [3.0–16.4] |  | 12/15 (80.0) |  | 21.2 [10.8–46.3] |  | 7/13 (53.8) |
| Moderate | 9.22 [3.9–51.3] |  | 20/23 (87.0) |  | 32.8 [10.8–78.5] |  | 14/22 (63.6) |
| Disease extent |  | 0.39 |  |  |  | 0.83 |  |
| Proctitis | 6.82 [3.1–15.2] |  | 5/7 (71.4) |  | 21.1 [8.7–64.8] |  | 4/7 (57.1) |
| Left-sided colitis | 4.67 [2.3–41.0] |  | 7/10 (70.0) |  | 29.6 [14.3–72.0] |  | 6/9 (66.7) |
| Pancolitis | 14.2 [4.2–41.4] |  | 20/21 (95.2) |  | 21.6 [9.1–72.6] |  | 11/19 (57.9) |
| Refractory UC |  | 0.31 |  |  |  | 0.43 |  |
| No | 8.86 [3.2–21.8] |  | 19/25 (76.0) |  | 39.4 [11.9–76.3] |  | 16/23 (69.6) |
| Yes | 7.95 [4.7–65.3] |  | 13/13 (100) |  | 15.6 [9.8–43.2] |  | 5/12 (41.7) |
| Extraintestinal complication | | 0.64 |  |  |  | 0.41 |  |
| No | 9.22 [3.6–50.3] |  | 24/29 (82.8) |  | 32.7 [11.0–88.4] |  | 16/26 (61.5) |
| Yes | 7.95 [3.9–14.2] |  | 8/9 (88.9) |  | 19.9 [7.2–62.3] |  | 5/9 (55.6) |

UC, ulcerative colitis; anti-αvβ6, anti-integrin αvβ6 antibody; anti-EPCR, anti-endothelial protein C receptor antibody; MFI, mean fluorescence intensity; IQR, interquartile range

**Supplementary Table 4. Longitudinal autoantibody titers between two time points**

1. **Before diagnosis (time points A and B)**

|  |  | A | B |
| --- | --- | --- | --- |
|  |  | −6.20 [−6.3–−5.1] years | −1.93[−2.6–−1.0] years |
| **anti-αvβ6** | Titer (U/ml, median[IQR]) | 1.48[1.05–41.1] | 20.3[1.46–78.0] |
|  | Positivity | 36.4% | 63.6% |
| **anti-EPCR** | Titer (MFI ratio, median[IQR]) | 17.8[9.80–34.9] | 45.8[36.4–109.8] |
|  | Positivity | 50.0% | 80.0% |

1. **Before and after diagnosis (time points C and D)**

|  |  | C | D |
| --- | --- | --- | --- |
|  |  | −2.19[−3.1–−1.5] years | +1.40[0.7–4.4] years |
| **anti-αvβ6** | Titer (U/ml, median[IQR]) | 2.96[1.35–30.6] | 9.85[1.68–43.1] |
|  | Positivity | 50.0% | 60.0% |
| **anti-EPCR** | Titer (MFI ratio, median[IQR]) | 19.2[12.4–61.1] | 49.3[22.2–128.3] |
|  | Positivity | 42.9% | 71.4% |

anti-αvβ6, anti-integrin αvβ6 antibody; anti-EPCR, anti-endothelial protein C receptor antibody; MFI, mean fluorescence intensity; IQR, interquartile range

**Supplementary Table 5. Logistic regression analysis for each nutrient**

|  | Cutoff points | | |  | Number of Cases | | | |  | OR(95%CI) | | | | Trend P |
| --- | --- | --- | --- | --- | --- | --- | --- | --- | --- | --- | --- | --- | --- | --- |
|  | Q1/Q2 | Q2/Q3 | Q3/Q4 |  | Q1 | Q2 | Q3 | Q4 |  | Q1 | Q2 | Q3 | Q4 |  |
| Protein (g) | 78.04 | 79.87 | 82.1 |  | 11 | 6 | 10 | 9 |  | (ref) | 0.54(0.18–1.42) | 0.91(0.37–2.18) | 0.82(0.32–2.00) | 0.89 |
| Fat (g) | 58.94 | 60.24 | 61.75 |  | 10 | 10 | 7 | 9 |  | (ref) | 1.0(0.40–2.48) | 0.69(0.25–1.82) | 0.90(0.35–2.28) | 0.66 |
| Carbohydrate (g) | 260 | 267.1 | 272.2 |  | 10 | 8 | 8 | 10 |  | (ref) | 0.79(0.30–2.04) | 0.79(0.30–2.05) | 1.00(0.40–2.48) | 1 |
| Potassium (mg) | 3,100 | 3,227 | 3,361 |  | 9 | 7 | 11 | 9 |  | (ref) | 0.77(0.27–2.1) | 1.24(0.50–3.11) | 1.00(0.38–2.60) | 0.76 |
| Calcium (mg) | 595 | 630 | 664 |  | 12 | 6 | 9 | 9 |  | (ref) | 0.48(0.17–1.28) | 0.74(0.30–1.79) | 0.74(0.30–1.78) | 0.65 |
| Iron (mg) | 8.76 | 9.11 | 9.67 |  | 7 | 13 | 5 | 11 |  | (ref) | 2.05(0.83–5.53) | 0.73(0.21–2.34) | 1.66(0.64–4.56) | 0.72 |
| Vitamin B6 (mg) | 0.92 | 1.11 | 1.17 |  | 6 | 12 | 11 | 7 |  | (ref) | 2.08(0.79–6.04) | 1.86(0.70–5.47) | 1.2(0.39–3.77) | 0.85 |
| Vitamin B12 (µg) | 8.16 | 8.88 | 9.45 |  | 10 | 8 | 5 | 13 |  | (ref) | 0.80(0.30–2.06) | 0.49(0.15–1.41) | 1.32(0.57–3.14) | 0.64 |
| Folic acid (µg) | 405 | 427 | 452 |  | 8 | 10 | 10 | 8 |  | (ref) | 1.26(0.49–3.35) | 1.26(0.49–3.36) | 1.00(0.36–2.76) | 1 |
| Vitamin C (mg) | 137 | 144 | 152 |  | 10 | 8 | 12 | 6 |  | (ref) | 0.79(0.30–2.04) | 1.21(0.51–2.92) | 0.59(0.20–1.61) | 0.55 |
| SFA (g) | 16.1 | 16.7 | 17.4 |  | 11 | 8 | 8 | 9 |  | (ref) | 0.93(0.36–2.34) | 0.82(0.31–2.04) | 0.92(0.36–2.25) | 0.78 |
| Dietary fiber (g) | 16.2 | 17.2 | 18.2 |  | 8 | 8 | 12 | 8 |  | (ref) | 1.0(0.36–2.76) | 1.52(0.62–3.94) | 1.0(0.36–2.77) | 0.76 |
| Salt (g) | 9.44 | 10.3 | 10.6 |  | 8 | 14 | 5 | 9 |  | (ref) | 1.79(0.75–4.55) | 0.62(0.19–1.88) | 1.13(0.43–3.05) | 0.65 |
| n-3 PUFA (g) | 2.03 | 2.23 | 2.36 |  | 13 | 8 | 9 | 6 |  | (ref) | 0.60(0.23–1.45) | 0.71(0.29–1.66) | 0.45(0.16–1.16) | 0.14 |
| n-6 PUFA (g) | 9.68 | 10.03 | 10.35 |  | 7 | 9 | 10 | 10 |  | (ref) | 1.29(0.47–3.64) | 1.46(0.55–4.08) | 1.44(0.55–4.03) | 0.44 |
| Isoflavone (mg) | 32.09 | 36.24 | 41.2 |  | 7 | 8 | 13 | 8 |  | (ref) | 1.15(0.41–3.31) | 1.91(0.77–5.15) | 1.15(0.41–3.31) | 0.54 |
| β-carotene (µg) | 4,097 | 4,589 | 5,118 |  | 12 | 7 | 8 | 9 |  | (ref) | 0.57(0.21–1.45) | 0.66(0.25–1.62) | 0.74(0.30–1.78) | 0.55 |

OR, odds ratio; CI, confidence interval; ref, reference; SFA, saturated fatty acid; PUFA, polyunsaturated fatty acids

**Supplementary Table 6. Logistic regression analysis for each food category**

|  | Cutoff points (g) | | |  | Number of Cases | | | |  | OR(95%CI) | | | | Trend P |
| --- | --- | --- | --- | --- | --- | --- | --- | --- | --- | --- | --- | --- | --- | --- |
|  | Q1/Q2 | Q2/Q3 | Q3/Q4 |  | Q1 | Q2 | Q3 | Q4 |  | Q1 | Q2 | Q3 | Q4 |  |
| Cereals | 371.2 | 394.6 | 417.8 |  | 10 | 7 | 8 | 11 |  | (ref) | 0.69(0.25–1.83) | 0.80(0.30–2.05) | 1.10(0.46–2.69) | 0.76 |
| Potatoes | 37.25 | 43.11 | 45.66 |  | 8 | 8 | 4 | 16 |  | (ref) | 1.0(0.36–2.76) | 0.49(0.13–1.59) | 2.06(0.89–5.16) | 0.13 |
| Nuts and seeds | 57.38 | 71.83 | 83.84 |  | 9 | 6 | 8 | 13 |  | (ref) | 0.63(0.21–1.76) | 0.94(0.35–2.49) | 1.47(0.62–3.61) | 0.26 |
| Vegetables | 315.2 | 342.1 | 367.9 |  | 11 | 7 | 8 | 10 |  | (ref) | 0.63(0.23–1.62) | 0.72(0.28–1.81) | 0.91(0.37–2.18) | 0.88 |
| Green-yellow vegetables | 137.3 | 151.5 | 167.1 |  | 10 | 5 | 11 | 10 |  | (ref) | 0.49(0.15–1.4) | 1.11(0.46–2.7) | 1.0(0.40–2.48) | 0.65 |
| Fruits | 139.7 | 157.9 | 175.5 |  | 9 | 7 | 12 | 8 |  | (ref) | 0.77(0.27–2.1) | 1.35(0.56–3.37) | 0.89(0.33–2.35) | 0.88 |
| Fungi | 12.8 | 16.8 | 22.2 |  | 6 | 11 | 10 | 9 |  | (ref) | 1.87(0.7–5.49) | 1.69(0.62–5.03) | 1.52(0.54–4.6) | 0.54 |
| Algae | 10.0 | 11.0 | 12.3 |  | 9 | 4 | 9 | 14 |  | (ref) | 0.44(0.12–1.37) | 1.0(0.39–2.62) | 1.59(0.69–3.88) | 0.13 |
| Fish and shellfish | 90.2 | 94.6 | 99.8 |  | 8 | 7 | 8 | 13 |  | (ref) | 0.87(0.3–2.45) | 1.0(0.37–2.78) | 1.66(0.69–4.24) | 0.22 |
| Meats | 65.8 | 75.6 | 90.3 |  | 10 | 10 | 9 | 7 |  | (ref) | 1.0(0.40–2.47) | 0.90(0.35–2.27) | 0.69(0.25–1.83) | 0.45 |
| Eggs | 32.1 | 34.0 | 35.4 |  | 7 | 9 | 9 | 11 |  | (ref) | 1.30(0.48–3.67) | 1.30(0.48–3.69) | 1.60(0.62–4.39) | 0.36 |
| Milk and dairy products | 124.4 | 154.4 | 176.9 |  | 9 | 10 | 9 | 8 |  | (ref) | 1.11(0.44–2.84) | 1.0(0.39–2.62) | 0.89(0.33–2.35) | 0.77 |

OR, odds ratio; CI, confidence interval; ref, reference
